# The Impact of Genetic Variation and Gene Expression Level of The Follicle-Stimulating Hormone Receptor on Ovarian Reserve

**DOI:** 10.22074/cellj.2018.4183

**Published:** 2017-11-04

**Authors:** Zeinab Ghezelayagh, Mehdi Totonchi, Shabnami Zarei-Moradi, Ommolbanin Asadpour, Saman Maroufizadeh, Poopak Eftekhari-Yazdi, Hamid Gourabi, Anahita Mohseni-Meybodi

**Affiliations:** 1 University of Science and Culture, Faculty of Basic Sciences and Advanced Technologies in Biology, ACECR, Tehran, Iran; 2 Department of Genetics, Reproductive Biomedicine Research Center, Royan Institute for Reproductive Biomedicine, ACECR, Tehran, Iran; 3 Department of Epidemiology and Reproductive Health, Reproductive Epidemiology Research Center, Royan Institute for Reproductive Biomedicine, ACECR, Tehran, Iran; 4 Department of Embryology, Reproductive Biomedicine Research Center, Royan Institute for Reproductive Biomedicine, ACECR, Tehran, Iran

**Keywords:** Allelic Variants, Follicle Stimulating Hormone Receptor, Premature Ovarian Failure

## Abstract

**Objective:**

Ovarian reserve is defined as the capacity of the ovary to provide fertile oocytes. Diminished ovarian reserve (DOR) is a disorder in which ovaries are prone to go through early menopause. Where this loss of function occurs before the age of 40, it results in the premature ovarian failure (POF) disease. Throughout folliculogenesis, the follicle-stimulating hormone receptor (FSHR) starts a signaling cascade in the granulosa cells where its inactivation leads to the arrest of follicle maturation and therefore adversely affects ovarian reserve. The aim of this study was to investigate the association of genetic variation (polymorphisms and inactivating mutations) of FSHR with POF and DOR.

**Materials and Methods:**

This case-control study comprised 84 POF, 52 DOR and 80 fertile Iranian women. To determine the presence of the 566C>T mutation and the -29G>A polymorphism in FSHR, PCR-RFLP method was used. SSCP-sequencing was used to identify any allelic variants in exon 10. The expression of human FSHR at the transcript level was also compared between DOR and fertile controls by real time-polymerase chain reaction (PCR).

**Results:**

The 566C>T polymorphism was normal in all the cases. All genotypes of -29G>A and 919G>A (exon 10) polymorphisms were observed. Statistically significant differences were seen in the genotypic distribution of both polymorphisms when comparing the control group with the DOR patient group. A decrease was observed in FSHR expression of DOR patients compared with the control group but was not significant.

**Conclusion:**

We conclude that the -29G>A and 919G>A polymorphisms in FSHR may be associated with DOR. Although these polymorphisms had significant differences at the genic level, no significant variation was found at the transcript level.

## Introduction

Diminished ovarian reserve (DOR) is defined as an intermediate state between normal reproductive physiology and premature ovarian failure (POF), and characterized by a decrease in the number or quality of oocytes. Women with this disorder, despite displaying a normal reproductive cycle, have high levels of the follicle-stimulating hormone (FSH) (1). POF, a gonad developmental defect with complete cessation of ovarian function, is a heterogeneous ovarian disorder affecting approximately 1% of women under the age of 40 (2). It is characterized by amenorrhea accompanied by high levels of gonadotropin hormones and low levels of estrogen in blood plasma (3). Follicles beyond the preantral stage are not developed in defective ovaries and since patients have high FSH levels in their blood serum, it suggests that the FSH receptor gene (FSHR) may be responsible for the observed functional defects (4, 5). The human FSHR is located on chromosome 2p21 (6). It produces a glycoprotein hormone receptor, a member of the G-protein-coupled-receptor family. Exons 1-9 encode the extracellular domain whilst all other domains including the intracellular, the transmembrane and the C-terminal of the extracellular domain are encoded by exon 10 (4).

Several inactivating mutations and polymorphisms have been identified in FSHR in women with primary and secondary amenorrhea. In 1995, the 566C>T (rs121909658) missense variant was the first reported for this gene detected in six Finnish families with hypergonadotrophic hypogonadism and early amenorrhea. Several other inactivating mutations and a few polymorphisms were reported afterwards (7-11). Furthermore, in 2011, a relationship between FSHR expression level and the genotype at the -29G>A (rs1394205, located in the promoter) polymorphism was observed in patients where a decrease in expression was associated with the AA genotype when compared with the GG genotype (12). In the present study, the presence of the 566C>T mutation in exon 7 and the -29G>A polymorphism in the promoter region of FSHR was investigated. As the intracellular, the transmembrane and the C-terminal of the extracellular domains of FSHR are encoded by exon 10, this exon was screened to detect novel allelic variants (Table 1). In addition, the level of human FSHR expression at the transcript level was compared between the DOR and the control groups.

## Materials and Methods

This case-control study comprised 84 POF patients, 52 DOR patients and 80 fertile women as the control group who had proven fertility, no history of irregular menstrual cycles, and normal serum FSH and luteinizing hormone (LH) levels. Patients in the DOR group were selected based on the Bolognia criteria which are 3 oocytes with a conventional stimulation protocol, antral follicle counts (AFC) 5-7 (2-10 mm in diameter, using standardized two-dimensional technique), FSH levels>11 IU/l at day 3 of the follicular cycle, under 40 years of age and regular menstrual cycles for the past 6 months. The selection criteria for the POF patient group was i. No history of either autoimmune disorder or surgery, ii. Normal 46,XX karyotype, iii. Serum FSH levels >40 IU/l at day 3 of the follicular cycle, iv. 6 months of amenorrhea and menses cessation, v. <40 years old and vi. Functional FMR1 which is one of the known causes of POF (13). All women were of Iranian origin. This study was approved by the Ethics Committee for Clinical Research at Royan institute and informed written consent was obtained from all participants. Additional clinical information was extracted from records of each patient.

### Polymorphism and mutation genotyping

Based on the database of polymorphisms with clinical significance (http://www.ncbi.nlm.gov/) and related articles (7, 8, 10, 14) which have investigated certain polymorphisms and inactivating mutations in POF and DOR patients, the promoter region, exon 7 and exon 10 of FSHR were selected for this study. The -29G>A polymorphism in the promoter region and the 566C>T mutation in exon 7 were genotyped by polymerase chain reaction-restriction fragment length polymorphism (PCR-RFLP) and exon 10 was screened by single-strand conformation polymorphism followed by sequencing (SSCP-sequencing) (Table 1).

### DNA extraction and polymerase chain reaction

Genomic DNA was extracted from peripheral blood leucocytes by the salting out method. The fragments harboring the selected SNPs were amplified by PCR with the use of specific oligonucleotide primers designed by Perl primer version1.1.20 (Table 1). PCR amplification was performed in a 25 μl reaction mixture containing 1.5 mM MgCl2, 0.2 mM dNTP, 1X PCR buffer, 0.06 U/μl Taq DNA polymerase enzyme (all from Cinagen, Iran), 0.4 pmol of each primer (Fazapajoh, Iran) and 2 μl of the DNA template. The PCR conditions for all except the promoter region SNP (the-29G>A polymorphism) were an initial DNA denaturation at 95˚C for 5 minutes, followed by 30 cycles of DNA denaturation at 95˚C for 45 seconds, annealing at melting temperature (TM) for 45 seconds (Table 1) and extension at 72˚C for 45 seconds followed by a final extension at 72˚C for 10 minutes. The PCR cycling conditions for the promoter SNP were 4 minutes of initial DNA denaturation at 94˚C followed by 30 cycles that consisted of 45 seconds of denaturation at 94˚C, 45 seconds of annealing at 64˚C and 45 seconds of extension at 72˚C followed by 8 minutes for final extension. All PCR reactions were performed in a master cycler gradient thermocycler (Eppendorf, Germany). All PCR products were run on a 1.7% ultra-pure agarose gel (Invitrogen, USA), stained by ethidium bromide (Invitrogen, USA) and visualized under the UV light.

**Table 1:** The sequence of primers used in this study


Fragment (Variant/ Ref number)	Primer sequence (5ˊ-3ˊ)	PCR product size (bp)	TM (°C)	Subsequent reaction	Restriction fragments (bp)	
					Mutant allele	Normal allele

Exon 10-A	F: AACTCATCATTTCTACCCTGCAC	396	61	SSCP	-	-
	R: GGATCACTAGCACTATGATGTTCC					
Exon 10-B	F: CTGCCAGTGTCATGGTGATG	239	60	SSCP	-	-
	R: AGAGGAGGACACGATGTTGG					
Exon 10-C	F: TTCTGCTGGTTCTGTTTCAC	324	61	SSCP	-	-
	R: TACCCTTCAAAGGCAAGACTG					
Exon 7 (566C>T/ rs121909658)	F: CCCGTGTATTGTTTGCATCTGA	182	59	RFLP (BsmI)	189	84/98
	R: CTGTTGTAAGAGCCATTTCCCT					
Promoter(-29G>A/ rs1394205)	F: ACCCTACCAGTTCTCAAGTCA	240	63	RFLP (BMOII)	18/222	18/84/138
	R: GAATCTCTGTCACCTTGCTCTC					


TM; Temperature of melting; PCR; Polymerase chain reaction, SSCP; Single-strand conformation polymorphism, RFLP; Restriction fragment length polymorphism.

### Restriction fragment length polymorphism

PCR products of exon 7 were digested with the BsmI enzyme (Fermentase, USA) for the 566C>T mutation. Digestion was performed in a 31 μl solution containing 1 μl restriction enzyme, 2 μl 10X buffer R (included along with the digestion enzyme), 18 μl diluted water and 10 μl of the PCR product. The solution was incubated for 16 hours at 37˚C. Digested fragments were separated by electrophoresis on an 8% polyacrylamide gel for 4 hours at 250 V and were visualized by staining with ethidium bromide. PCR products of the promoter region were digested with the MBOII enzyme (Fermentase, USA) for the -29G>A polymorphism. Digestion was performed in a 20 μl solution containing 0.3 μl restriction enzyme, 2 μl buffer (included along with the digestion enzyme), 7.7 μl diluted water and 10 μl of the PCR product, and incubated for 2 hours at 37˚C. The digested fragments were separated using a 2% ultra-pure agarose gel and visualized by staining with ethidium bromide.

### Single-strand conformation polymorphism

All PCR products of exon 10 were screened by SSCP. Briefly, 3 μl of the PCR products were mixed with 8 μl of the formamide dye (Roche, France) and denatured at 95˚C for 10 minutes before being transferred onto ice. They were then electrophoresed at 150 V for 16-20 hours on an 8% polyacrylamide gel. The gels were visualized by silver staining (Sigma, Iran).

### Sequencing

To ensure the validity of the RFLP results, about half of the RFLP PCR products and 10 samples of each of the differential migration patterns observed in SSCP were subsequently sequenced by an ABI automated DNA sequencer (Macrogen, Korea). The results were analyzed by Finch TV software version 1.4.0 (http://www.geospza.com/products/finch TV.shtml) and were compared with the database reference sequence (http://www.ebi.ac.uk/tools/sss).

### Data and statistical analysis

Quantitative variables were expressed as mean ± SD and categorical variables were expressed as frequencies (in percentage). The difference in genotypic distribution and the variation of allele frequencies in the control and patient groups were examined using a Chi-square test. All statistical analyses were conducted in SPSS version 16.0 (SPSS Inc., Chicago, IL, USA) and a P<0.05 was considered statistically significant.

### RNA extraction and quantitative reverse transcriptasepolymerase chain reaction

The granulosa cells of 16 DOR patients were used to study FSHR expression. To accomplish this, DOR patients (n=8) with high FSH blood level and rare follicles (≤3) were categorized as the case group and the control group (n=8) included patients with low FSH blood level and more follicles (>3). Total RNA was extracted from granulosa cells using the Absolutely RNA Nanoprep kit (Aligent, USA) in accordance with the manufacturer’s instructions. The integrity of total RNA was checked by denaturing formaldehyde/MOPS/1% agarose electrophoresis. The purity was also checked by UV-spectrophotometry in 10 mM Na_2_HPO_4_/NaH_2_PO_4_- buffer (pH=7.0). The A_260_/A_280_-ratio was larger than 2.0 and thus of sufficient purity. Two distinct ribosomal RNA bands were identified in each examined sample. A DNAse treatment to remove genomic DNA was carried out with RNAse-Free DNAse. RNA was then reverse transcribed by QuantiTect Whole Transcriptome kit (Qiagen, USA). To exclude possibility of genomic amplification, the PCR was also performed with the same total RNA samples but with no reverse transcriptase. All products were analyzed on a 4% agarose gel.

One Step Quantitative RT-PCR was performed on a 7500 Real time PCR system (Applied Bio System-USA) using SYBR Green. All reactions were run in triplicate to ensure consistency. In order to minimize the experimental error, all the stages except RNA extraction were repeated twice. Temperature profile of the real time-PCR consisted of 95˚C for 4 minutes, 40 cycles of 95˚C for 10 seconds and 60˚C for 30 seconds. The FSHR amplicon was a 156 bp inter-exonic product spanning exon 3 and 4:

F: ATTCCTTCTGACCTCCCGA
  R: GAACACATCTGCCTCTATCACC

ACTB was used as an internal control:

F: TCCCTGGAGAAGAGCTACG
  R: GTAGTTTCGTGGATGCCACA

The 2^-ΔΔCT^ was calculated to assess differential expression. The REST384 beta software (2006) was used to compare mean values between groups.

## Results

For each SNP examined, the genotypic distribution in the control group significantly deviated from the Hardy- Weinberg equilibrium (HWE). The main reason for this may be population stratification in the fertile control group. Genotyping error is unlikely since no deviation was observed in the POF and DOR groups. The clinical data of the patients are shown in Table 2.

### Polymorphism and mutation genotyping results

#### Restriction fragment length polymorphism results

**Fig.1:**
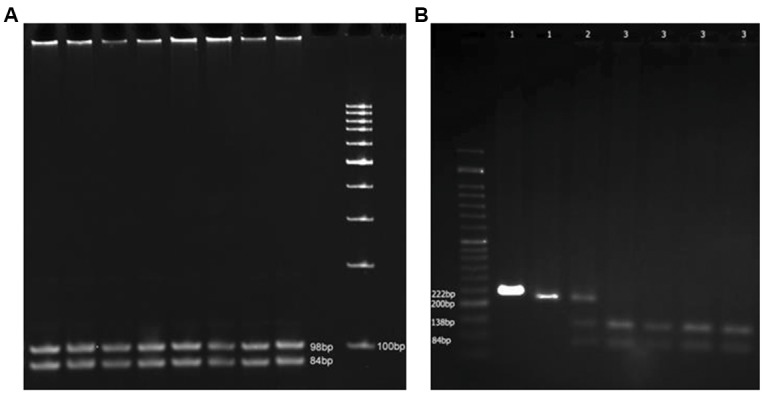
Polymerase chain reaction (PCR) products after enzymatic digestion. A. Enzymatic digestion of PCR products harboring the 566C>T mutation by BsmI enzyme. Since all the samples are digested, none carry the T allele and B. Enzymatic digestion of PCR products harboring the -29G>A polymorphism by BMOII enzyme. A 50 bp ladder is used. The 18 bp band is not noticeable. Lane 1; Mutant samples, Lane 2; Heterozygote sample, and Lane 3; Wild type samples.

All the PCR reactions had product sizes as expected. The 189 bp product harboring the 566C>T mutation (exon 7) produced two fragments of 98 and 84 bp after digestion in all samples, indicating that no homozygote or heterozygote mutation was present (Fig .1A). For the promoter SNP, 69.2% of controls, and 58.5% of POF and 46.2% of DOR patients were wild type (GG). 20.5% control, 31.7% POF and 46.2% DOR patients had heterozygote alleles (GA) and 10.3%, 9.8% and 7.7% were mutant (AA), respectively (Fig .1B). This difference was statistically significant (P=0.04) among the studied groups (Table 3). This difference was significant when only comparing the DOR patients with the control group (P=0.008) whilst no significant association (P=0.268) was seen when comparing only with the POF group.

**Table 2:** Comparison of clinical data between POF and DOR patients


Patients		Age mean (Y)(mean ± SD)	Menarche age mean (Y)(mean ± SD)	FSH mean (IU/L)(mean ± SD)	LH mean (IU/L)(mean ± SD)	Amenorrhea age mean (Y)(mean ± SD)

POF						
	Primary amenorrhea	29.73 ± 5	17.29 ± 3.3	62.67 ± 6.1	20.37 ± 1.6	-
	Secondary amenorrhea	31.17 ± 3.8	13.17 ± 1.2	73.96 ± 3.2	35.95 ± 2.2	23.6 ± 4.3
DOR		34.38 ± 6.8	13.18 ± 1.6	9.56 ± 5.8	2.85 ± 1.6	-


POF; Premature ovarian failure, DOR; Diminished ovarian reserve, FSH; Follicle-stimulating hormone, and LH; Luteinizing hormone.

**Table 3:** The distribution of the -29G>A genotypes observed in the studied groups


-29G>A genotypes	Count per group (%)		
	Controln (%)	POFn (%)	DORn (%)

Wild type	54 (69.2%)	48 (58.5%)	24 (46.15)
Heterozygote	16 (20.5%)	26 (31.7%)	24 (46.15)
Full mutation	8 (10.3%)	8 (9.8%)	4 (7.7)
Total	78	82	52


The difference between wild type, heterozygote and full mutation was significant (P=0.04) among the 3 groups and also between the DOR patients with the control group (P=0.008, statistical test: Chi-square test). POF; Premature ovarian failure and DOR; Diminished ovarian reserve.

### Single-strand conformation polymorphism results

**Fig.2:**
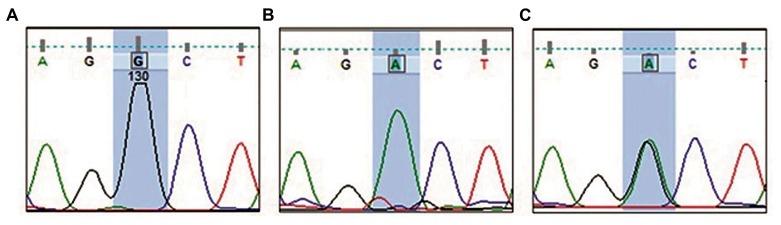
Partial electropherogram from DNA sequencing of polymerase chain reaction (PCR) products. A. A 919A (Ala at position 307) indicating the wild type sequence, B. T919T (Thr at position 307) indicating the mutant sequence, and C. A919T (Ala and Thr at position 307) indicating the heterozygote sequence.

No abnormal SSCP migration pattern was observed in the PCR products of exon 10. The SSCP results were analyzed and confirmed by direct sequencing and the 919G>A common SNP which corresponds to the amino acid substitution Ala307Thr was identified (Fig .2). The distribution of 919G>A genotypes are given in Table 4. The difference in genotypic distribution was statistically significant among the studied groups (P=0.007) and also when only comparing the DOR group with the control group (P=0.008). No other variants were observed in exon 10 in all the samples.

### Expression and real time results

The results indicated that the FSHR transcript was expressed in granulose cells of both the control and DOR groups. Although the FSHR gene expression rate in DOR patients were lower than that of the control group, it was not statistically significant (P>0.05).

**Table 4:** The distribution of the 919G>A genotypes observed in the studied samples


919G>A genotypes	Count per group (%)		
	Controln (%)	POFn (%)	DORn (%)

Wild type	28 (35%)	24 (%28.6)	6 (%11.5)
Heterozygote	48 (60%)	50 (59.5%)	44 (%84.6)
Full mutation	4 (5%)	10 (11.9%)	2 (%3.8)
Total	80	84	52


The difference between wild type, heterozygote and full mutation was significant (P=0.07) among the 3 groups and also between the DOR patients with the control group (P=0.008, statistical test: Chi-square test). POF; Premature ovarian failure and DOR; Diminished ovarian reserve.

## Discussion

Development and maturation of ovarian follicles depends on the interaction of FSH with its receptor, which in turn is essential for female fertility. Any variants in FSHR that decreases either the interface between FSHR and its ligand or the transmission of its signal after connection may lead to the decrease in ovarian reserve. Mutations in the extracellular domain results in a complete inability of the receptor for hormone connection or blocks signal transfer after hormone binding (8, 15). Variants located within the transmembrane domain may be involved in the proper placement of the receptor in the membrane. It is thus expected that inactivating mutations in the intracellular domain may impair intracellular signaling.

Although DOR is more common than POF (10% compared to nearly 1%), fewer studies have been conducted on the former. To the best of our knowledge, this research is one of the few studies investigating the allelic variants of this gene in DOR patients (16). The mutations studied so far, especially in poor responders, were -29G>A, 566C>T, 919A>G, and 2039A>G (16-19). In the present study no variants were identified except for the two common polymorphisms, -29G>A and 919A>G. Reports from India demonstrates that the AA genotype at position -29 and the Asn/Asn at position 680 are both correlated with poor response to gonadotropin treatment (17, 18). In the present study, the frequency distribution of -29G>A genotypes was significantly different between the control and DOR groups (P=0.008). Livshyts et al. (19) showed that the presence of 919A>G and 2039A>G polymorphisms together were associated with diminished reserve in Ukrainian patients, which is similar to the results reported here for the 919A>G polymorphism (DOR vs. control).

In 1995, the 556C>T mutation was the first inactivating mutation detected which showed a correlation between FSHR and POF in the Finnish population (10). Although Jiang et al. (20) identified only one mutation carrier in a large-scale screening study of the Swiss population, a strong enrichment of this mutation was shown in the northeastern part of Finland with a frequency of 0.96%. Other studies in diverse populations have shown the absence of this mutation (16, 21, 22) which is in agreement with its absence in POF patients. It is thus likely that this mutation is restricted to Finland which may represent a founder effect in this region. No other variants were found in studies conducted in England (22), Argentina (21), Brazil (23), India (9), Singapore (24) and in Iranian POF patients of this study.

We detected no significant difference between the allelic distribution of the -29G>A SNP between the control group and the POF group, however, in the Indian population the frequency of the AA genotype was significantly higher in primary and secondary amenorrhea (9). The 919A>G polymorphism has been widely studied in a variety of infertility disorders in different populations (24-26). In the present study, although the 919A>G polymorphism was observed among POF patients, there was no significant association with this SNP. Several other studies in different populations equally showed no such association with this disease (22, 25, 27). In a study conducted in Brazil, a significant association between the age of amenorrhea onset and genotype of the 919A>G polymorphism was observed but has yet to be confirmed (23). Due to the small number of secondary amenorrhea patients in our study, no relationship between these two factors was observed.

Since it is difficult to obtain granulosa cells from POF patients, no studies associated with FSHR expression in these patients have been performed so far. Cai et al. (28) found an association between the low expression level of FSHR and poor ovarian response to gonadotropin stimulation while Desai et al. (12) showed a relationship between the AA genotype at the -29G>A SNP and this lower expression of the receptor on the granulosa cells in poor ovarian response patients. In contrast, Wunsch et al. (29) found no correlation between ovarian response and this polymorphism in German and Indonesian populations. The results of the present study showed a decrease in the transcript expression of FSHR in DOR patients compared with the control group. This decrease was not statistically significant which may be due to the lower sample size in this study.

## Conclusion

We conclude that among all the polymorphisms studied, only the 919A>G and the -29G>A polymorphisms of FSHR may predispose an individual to the depletion of the ovarian reserve but given the deviation of genotype frequencies in the control group from HWE, caution must be taken with this conclusion. However, the introns and other exons of this gene must be studied to confirm this preliminary finding. Since the non-significant change in FSHR expression may be due to the low sample size, its expression should be studied in a larger Iranian population as well as other populations to examine this association further.
